# Can the Glasgow prognostic score predict ischemic stroke in patients with infective endocarditis?

**DOI:** 10.1590/1806-9282.20231299

**Published:** 2024-04-22

**Authors:** Cihan Aydın, Aykut Demirkıran, Hüseyin Aykaç, Nurullah Uslu, Şeref Alpsoy

**Affiliations:** 1 Namık Kemal University, Faculty of Medicine, Department of Cardiology – Tekirdağ, Turkey.

**Keywords:** Prognosis, Transesophageal echocardiography, C-reactive protein, Albumin

## Abstract

**OBJECTIVE:**

The Glasgow prognosis score is a simple parameter calculated using serum levels of albumin and C-reactive protein. The aim of this study was to examine whether this parameter may predict ischemic stroke in patients with infective endocarditis.

**METHODS:**

A total of 80 patients who were diagnosed with definitive infective endocarditis according to Duke criteria between 2016 and 2023 were included in the study. Glasgow prognosis score was based on serum levels of albumin and C-reactive protein. In imaging methods, patients were divided into two groups according to whether they had a stroke or not. These two groups were compared in terms of biochemical parameters, and infective endocarditis findings on echocardiography and Glasgow prognosis score.

**RESULTS:**

We found that the results were statistically similar except for serum C-reactive protein (Group 1: 54.9±71.1 and Group 2: 39±70.7; p=0.03), neutrophil (Group 1: 19.8±10.8*10^9^/L and Group 2: 13.3±7.3*10^9^/L; p=0.014), albumin (Group 1: 2.3±0.6 and Group 2: 2.8±0.5; p=0.03), and Glasgow prognosis score (Group 1: median 2, min.–max. (1–2) and Group 2: median 1, min.–max. (0–1); p=0.004). In the receiver operating characteristics analysis, Glasgow prognosis score had 82.4% sensitivity and 58.3% specificity in predicting ischemic stroke if the Glasgow prognosis score cutoff was ≥1. In multivariate logistic regression analysis, chronic renal failure [odds ratio (OR): 1.098; 95% confidence interval: 1.054–1.964; p=0.044], age (OR: 1.050; 95%CI 1.006–1.096; p=0.024), and Glasgow prognosis score (OR: 0.695; 95%CI 0.411–0.949; p=0.035) were independent variables in predicting ischemic stroke.

**CONCLUSION:**

High Glasgow prognosis score is an independent predictor of ischemic stroke in patients with infective endocarditis. Glasgow prognosis score, determined using albumin and C-reactive protein levels, is a simple and practical index for predicting the prognosis of patients hospitalized with infective endocarditis.

## INTRODUCTION

Although infective endocarditis (IE) is uncommon, it is a serious condition with a mortality rate of 12–20% during the initial hospitalization^1^. Mortality rates during hospitalization are around 3%^2^. Several characteristics are frequently reported as indicators of poor prognosis: older age, heart failure, renal failure, staphylococci infection, aortic location, embolisms, IE on the prosthetic valve, and persistent fever despite antibiotic therapy^3^. New prognostic markers are needed to predict high-risk IE patients. Many studies have shown that inflammation plays an important role in the etiopathogenesis of cardiovascular diseases^4^.

The Glasgow prognostic score (GPS), calculated from C-reactive protein (CRP) and albumin levels, is a useful tool in predicting prognosis in various cancer types^5^. In addition, GPS has been stated in various studies as an important parameter in predicting survival in heart failure with reduced ejection fraction and preserved ejection fraction and predicting mortality in patients with acute coronary syndrome and IE^5-7^. When accompanied by cerebral embolism, IE, an uncommon illness, has significant morbidity and mortality. We aimed to examine whether this parameter can predict ischemic stroke in patients with IE.

## METHODS

### Study population

A total of 80 patients who were diagnosed with definite IE according to Duke criteria between 2016 and 2023 were included in the study retrospectively. However, 20 patients with carotid artery disease detected on Doppler ultrasonography and 10 patients with missing laboratory results were excluded from the study. There were no signs of cerebrovascular embolism in the physical examination and imaging methods of the patients with IE included in the study at the time of admission to the hospital. Patients who developed stroke during hospital follow-up were included in Group 1. Patients who did not develop stroke were included in Group 2. Patient data was obtained from the data system of our hospital. This study was carried out at Tekirdağ Namık Kemal University Hospital, Department of Cardiology. The study protocol was reviewed and approved by the institutional ethics committee (Ethics Committee Number: 2023.150.07.15) and by the principles of the Declaration of Helsinki. Informed consent was obtained from all the patients participating in the study. In exclusion criteria, patients younger than 18 years of age without knowledge of serum albumin and/or CRP levels, patients with other increased inflammatory markers such as malignancy, patients receiving systemic steroid therapy, patients with chronic inflammatory disease, and patients with end-stage liver disease were excluded from the study. Patients with carotid artery disease in Doppler ultrasonography were excluded from the study. Carotid artery stenosis was referred to as a≥50% stenosis of internal carotid artery, with stenosis severity estimated using the North American Symptomatic Carotid Endarterectomy Trial method^8^.

### Collection of blood samples and laboratory measurement

Basic demographic and clinical variables of the study population, such as medical history, physical examination, age, gender, diabetes mellitus, hypertension, dyslipidemia, and smoking, were recorded from details in the hospital database. Blood tests and GPS were calculated based on the blood values at the time of admission to the hospital. Three sets of blood cultures (six vials in total, three aerobic and three anaerobic) were taken at half-hour intervals, without waiting for the febrile period to occur in the patients. GPS was calculated as follows: patients with both normal CRP (≤1.0 mg/dL) and albumin (≥3.5 mg/dL) levels were given 0 points. One point was given to patients with abnormal CRP or abnormal albumin levels. Patients with both high CRP (>1.0 mg/dL) and hypoalbuminemia (<3.5 mg/dL) were given 2 points^9^. Blood culture positivity and accompanying microorganisms were recorded.

### Clinical follow-up

Transthoracic echocardiography and/or transesophageal echocardiography (TEE) were performed with the Epiq 7 device (Koninklijke Philips N.V., Amsterdam, the Netherlands). Intracardiac complications such as vegetation presence, valve type, left ventricular dysfunction, perivalvular abscess, leaflet perforation, paravalvular regurgitation, and new prosthetic valve regurgitation were evaluated, and the results were recorded. Findings other than sinus rhythms were determined from electrographic recordings. Clinical complications such as acute heart failure, acute renal failure, peripheral embolism, the need for surgery, and death were recorded. Newly developed stroke events and septic embolisms were detected using cranial computed tomography (CT) and cranial magnetic resonance imaging (MRI) methods.

### Statistical analysis

Statistical analysis was performed using the SPSS 22.0 statistics package (SPSS Inc., Chicago, IL, USA). Categorical variables were expressed as percentages. The chi-square test and Fisher's exact tests were used for categorical variables. Normally distributed data were reported as mean±standard deviation after being analyzed with the Kolmogorov–Smirnov test, while non-normally distributed continuous variables were presented as median. The Student's t-test was used to compare normally distributed data, and the Mann–Whitney U test was used to compare non-normally distributed data. Univariate and multivariate logistic regression analyses were used to determine the independent predictors of stroke. Receiver operating characteristic (ROC) analysis was performed to determine the optimal cutoff value of GPS to predict stroke. A p<0.05 was considered statistically significant.

## RESULTS

A total of 80 patients diagnosed with IE, according to Duke's criteria, were included in our study. These patients were divided into two groups: those who were found to have had a stroke as a result of cranial MRI, CT, and physical examination (Group 1, n=23) and those who did not have a stroke (Group 2, n=57). Out of these 80 patients, 49 (61.3%) were male, and the mean age was 66.7±1.6 years. Tables 1 and 2 describe the main demographic, laboratory, and clinical data of the groups. When we examined the basic laboratory and demographic characteristics of the patients, Group 1 was older (mean age, Group 1: 74.4±11.9 years vs. mean age, Group 2: 63.5±15.3 years; p=0.03), and the number of patients with atrial fibrillation (AF) [Group 1: 9 (39.1%) vs. Group 2: 4 (7%); p<0.001] was higher in Group 1. When the groups were examined in terms of laboratory parameters, white blood cell and neutrophil, creatinine, and blood urea nitrogen levels were higher in the stroke group. While GPS levels were higher in Group 1, albumin levels were lower in this group. The number of chronic renal failure patients was higher in the stroke group [Group 1: 18 (78.2%) vs. Group 2: 27 (47.3%); p=0.037]. The two groups were similar in terms of other demographic and laboratory parameters. While there was vegetation on the native mitral valve in 50 (62.5%) patients, vegetation on the native aortic valve was observed in 32 (40%) patients. In Group 1, the vegetation size of both mitral and aortic valves was larger than in Group 2. The two groups were similar in terms of complications after IE and surgical needs after complications. However, 34 patients died in their intensive care unit. In the ROC analysis, GPS had 82.4% sensitivity and 58.3% specificity in predicting ischemic stroke if the GPS was ≥1. The area under the curve was 0.625 [95% confidence interval (CI): 0.470–0.780; p=0.002]. In multivariate logistic regression analysis, chronic renal failure [odds ratio (OR): 1.098; 95%CI 1.054–1.964; p=0.044], age (OR: 1.050; 95%CI 1.006–1.096; p=0.024), and GPS (OR: 0.695; 95%CI 0.411–0.949; p=0.035) were independent predictors of stroke (Table 3).

**Table 1:** Demographic and laboratory variables of patients.

	All patients (n=80)	Group 1 (n=23)	Group 2 (n=57)	p-value
Age (years)	66.7±1.6	74.4±11.9	63.5±15.3	**0.031**
Gender (male), n (%)	49 (61.3)	11 (47.8)	38 (66.7)	0.117
Height (cm)	167.9±7.4	166.3±7.3	168.6±7.4	0.210
Weight (kg)	75.6±13.6	78.6±14.4	74.4±13.1	0.208
Hospitalization time (days)	26±19	27±20	25±18	0.760
Diabetes mellitus, n (%)	29 (36.3)	10 (43.5)	19 (33.3)	0.393
Hypertension, n (%)	60 (75)	20 (87)	40 (70.2)	0.117
Coronary artery disease, n (%)	34 (43)	10 (45.5)	24 (42.1)	0.788
Congestive heart failure, n (%)	14 (17.5)	5 (21.7)	9 (15.8)	0.860
Atrial fibrillation, n (%)	13 (16.2)	9 (39.1)	4 (7)	**<0.001**
Chronic renal failure, n (%)	45 (56.3)	18 (78.2)	27 (47.3)	**0.037**
Chronic obstructive pulmonary disease, n (%)	7 (8.7)	2 (8.6)	5 (8.7)	0.655
Angina, n (%)	18 (22.5)	5 (21.7)	13 (22.8)	0.918
Dyspnea, n (%)	43 (53.7)	10 (43.4)	33 (57.9)	0.226
Syncope, n (%)	6 (7.5)	2 (8.6)	4 (7)	0.232
Atrioventricular block	4 (5)	1 (4.3)	3 (5.2)	0.113
Hemoglobin (g/dL)	8.9±1.9	8.4±1.6	9.2±2	0.105
White blood cell (×10^3^/mm^3^)	18.6±9.3	23.7±10.3	16.5±8.1	**0.002**
Neutrophil (10^9^/L)	15.7±8.8	20.2±10.4	13.9±7.4	**0.003**
Thrombocyte (×10^3^/mm^3^)	161±10	151.6±7.8	166 ±10.9	0.567
C-reactive protein (mg/L)	40.6±6.3	56.3±6.8	34.3±6.3	0.160
Blood urea nitrogen (mg/dL)	125±34	164±72.9	104.6±71	**0.001**
Creatinine (mg/dL) (min.–max.)	2.7 (0.5–13.5)	4.78 (0.7–6)	1.7 (0.5–3.6)	0.067
Sodium (mmol/L)	134.3±7.43	134.5±9.6	134.41±5.8	0.959
Potassium (mmol/L)	4.7±1.4	4.4±1.1	4.7±0.5	0.937
Alanine aminotransferase (U/L) (min.–max.)	36 (6–580)	28 (6–580)	36 (9–200)	0.430
Aspartate aminotransferase (U/L) (min.–max.)	40 (10–636)	75 (10–721)	32 (12–400)	0.376
Albumin (g/dL)	2.7±0.6	2.3±0.6	2.9±0.5	**<0.001**
Glasgow prognostic score (min.–max.)	2 (0–2)	2 (1–2)	1 (0–2)	**<0.001**

Statistically significant values are denoted in bold. Group 1: patients with stroke; Group 2: patients without stroke. Chronic renal failure was defined as a creatinine value greater than 2 mg/dL.

**Table 2:** Echocardiographic variables of patients.

		All patients (n=80)	Group 1 (n=23)	Group 2 (n=57)	p-value
Ejection fraction (%)		56.4±7.1	55.9±7.5	56.5±7	0.727
SPAP (mmHg)		33.4±12.2	30±8	34±13.2	0.195
Mitral valve, n (%)					
	Native	77 (96.2)	22 (95.6)	55 (96.4)	0.847
	Prosthesis	3 (3)	1 (4)	2 (3)	0.113
Aortic valve, n (%)					
	Native	71 (88.7)	18 (78.2)	53 (92.9)	0.692
	Prosthesis	9 (11.3)	5 (21.7)	4 (7)	0.059
TEE evaluation, n (%)					
	Aortic vegetation	32 (40)	6 (26.1)	26 (45.6)	0.107
	Mitral vegetation	50 (62.5)	19 (82.6)	31 (54.4)	**0.018**
	Tricuspid vegetation	3 (3.7)	1 (4.3)	2 (3.5)	0.192
	Abscess	3 (3.7)	1 (4.3)	2 (3.5)	0.192
	Fistula	1 (1.25)	1 (4.3)	0 (0)	0.245
	Perforation	2 (2.5)	1 (4.3)	1 (1.7)	0.198
	Pseudoaneurysm	5 (6.2)	2 (8.6)	3 (5.2)	0.858
	Paravalvular leakage	5 (6.2)	2 (8.6)	3 (5.2)	0.125
	Prosthetic valve dehiscence	3 (25)	1 (16.6)	2 (33.3)	0.363
	Patients requiring surgery	13 (16.2)	4 (17.3)	9 (15.7)	0.287
Vegetation size (cm)					
	Aortic valve	0.34±0.19	0.36±0.17	0.17±0.05	**0.009**
	Mitral valve	0.73±0.6	0.99±0.77	0.62±0.61	**0.028**

Statistically significant values are denoted in bold. Group 1: patients with stroke; Group 2: patients without stroke. SPAP: systolic pulmonary artery pressure; TEE: transesophageal echocardiography.

**Table 3:** Univariate and multivariate logistic regression analysis of independent predictors of stroke.

Variables	Univariate analysis		Multivariate analysis	
	OR (95%CI)	p-value	OR (95%CI)	p-value
Age	1.058 (1.017–1.100)	**0.005**	1.050 (1.006–1.096)	**0.024**
Hypertension	2.833 (0.742–10.816)	0.128	–	–
Chronic renal failure	1.745 (1.307–2.452)	**0.039**	1.098 (1.054–1.964)	**0.044**
Heart failure	1.123 (0.308–4.087)	0.861	–	–
Glasgow prognostic score	2.509 (1.038–6.064)	**0.041**	0.695 (0.411–0.949)	**0.035**
Diabetes mellitus	1.538 (0.571–4.146)	0.394	–	–
Gender (female)	2.182 (0.814–5.850)	0.121	–	–
Atrial fibrillation	0.417 (0.043–4.025)	0.449	–	–

Statistically significant values are denoted in bold.

## DISCUSSION

Stroke is the third leading cause of death and disability in the world^10,11^. To the best of our knowledge, our study is one of the first studies in the literature to show a relationship between GPS and stroke in patients with IE. Previous studies have shown that long-term survival is significantly reduced in patients with high GPS and that chronic renal failure is an independent predictor of mortality^7^. In our study, we found age, chronic renal failure, and GPS as independent predictors of stroke. Inflammation plays an important role in the etiopathogenesis of cardiovascular diseases.

Inflammatory markers are associated with poor prognosis in cardiovascular diseases^8,9^.

Glasgow prognostic score includes two markers: negative acute phase reactant albumin and positive acute phase reactant CRP. In addition to its role in regulating osmotic pressure, albumin is a good indicator of nutritional status and has antioxidant and anti-inflammatory properties^12^. Hypoalbuminemia as a result of increased inflammation is a strong predictor of mortality in cardiovascular disease^13^. Furthermore, hypoalbuminemia is related to coagulation factors and is a prothrombotic condition^14^. The change in the thrombotic state may increase the frequency of stroke and peripheral embolism in IE. The most common complications in IE are embolic events (20.6%), acute renal failure (17.7%), and heart failure (14.1%)^15^.

C-reactive protein is an acute-phase reactant and a well-known marker of systemic inflammation. The relationship between high CRP levels and AF has been determined in previous studies. As it is known, one of the most important causes of stroke is AF^16^. Although the frequency of AF was low (16.2%) in our study, this rate was higher in the stroke group (39.1%).

All patients with AF were using oral anticoagulation therapy at the time of admission to the hospital. Their treatments were not changed during hospitalization. Additionally, TEE was performed in all our study patients, and no intracardiac thrombus was detected in any of the patients. Therefore, new cerebrovascular events were not attributed to AF.

Glasgow prognostic score was found to be an independent risk factor for 30-day and 1-year cardiovascular death in individuals with chronic coronary syndrome. Previous studies have revealed that the GPS score can identify a patient who is in poor condition with many diseases^17^. In many studies, GPS has also been reported to be associated with mortality and morbidity in cancer patients and in patients with heart failure with preserved and reduced ejection fraction^18,19^. The role of inflammation in the development of valve diseases is not to be underestimated. Various studies have shown that GPS can predict mortality after transcatheter aortic valve implantation (TAVI) and that low albumin levels prolong intensive care unit stays after TAVI, and high CRP levels increase the need for surgical repair in aortic valve stenosis^20-23^.

Infective endocarditis was complicated by a stroke in 20–40% of cases^24^. In our study, this rate was around 28%. Stroke is an independent adverse prognostic factor for survival in IE. Prompt initiation of antibiotic therapy in IE reduces the risk of stroke. Vegetation size (≥10 mm), mitral valve involvement, mobile vegetation, and *Staphylococcus aureus* infection have all been identified as risk factors for embolism. *S. aureus* grew in 25% of the patients in our study, and there was no difference between the two groups in terms of microorganisms grown in blood culture. Vegetation diameters were larger in the aortic and mitral valves in the stroke group.

In the stroke group, 82% of vegetation was on the mitral valve. Surgical requirements were also similar in the two groups. Individuals with chronic renal disease are more susceptible to complications such as malnutrition, cardiovascular events, anemia, and infections.

In our study, 56.3% of the patients had renal failure. This rate was higher in the stroke group (78.2%). Valve diseases are observed more frequently in renal failure due to impaired excretion of calcium and phosphate. When IE develops in the elderly and patients with kidney failure, it is difficult to maintain an effective antibiotic level. In our study, renal failure and age were found to be independent risk factors for stroke. Biomarkers such as GPS can help us determine the risk of stroke in these patients and guide the treatment strategy.

### Limitations

Our study was a retrospectively designed single-center study with a small number of patients. The patients were not inquired about using aspirin, statins, or anti-inflammatory medications earlier which would have affected the inflammatory process. GPS was calculated once from the patients’ laboratory values at the time of admission. GPS could be calculated from the average of all laboratory values that could be checked during follow-up.

Larger, multi-center prospective studies are needed to validate the findings of this work.

## CONCLUSION

High GPS is an independent predictor of ischemic stroke in patients with IE. GPS, determined using albumin and CRP levels, is a simple and practical index for predicting the prognosis of patients hospitalized with IE. These biomarkers may be a guide in determining patient risk before radiological imaging.
